# A 78 Seconds Complete Brain MRI Examination in Ischemic Stroke: A Prospective Cohort Study

**DOI:** 10.1002/jmri.28107

**Published:** 2022-02-16

**Authors:** Siri af Burén, Annika Kits, Lucas Lönn, Francesca De Luca, Tim Sprenger, Stefan Skare, Anna Falk Delgado

**Affiliations:** ^1^ Department of Radiology Capio Saint Göran Hospital Stockholm Sweden; ^2^ Department of Clinical Science, Intervention and Technology Karolinska Institute Stockholm Sweden; ^3^ Department of Clinical Neuroscience Karolinska Institute Stockholm Sweden; ^4^ Department of Neuroradiology Karolinska University Hospital Stockholm Sweden; ^5^ MR Applied Science Laboratory Europe, GE Healthcare Stockholm Sweden

**Keywords:** fast imaging, multicontrast MRI, EPIMix, ischemic stroke, brain infarction, diagnostic performance

## Abstract

**Background:**

Fast 78‐second multicontrast echo‐planar MRI (EPIMix) has shown good diagnostic performance for detecting infarctions at a comprehensive stroke center, but its diagnostic performance has not been evaluated in a prospective study at a primary stroke center.

**Purpose:**

To prospectively determine whether EPIMix was noninferior in detecting ischemic lesions compared to routine clinical MRI.

**Study type:**

Prospective cohort study.

**Population:**

A total of 118 patients with acute MRI and symptoms of ischemic stroke.

**Field Strength and Sequence:**

A 3 T. EPIMix (echo‐planar based: T1‐FLAIR, T2‐weighted, T2‐FLAIR, T2*, DWI) and routine clinical MRI sequences (T1‐weighted fast spin echo, T2‐weighted PROPELLER, T2‐weighted‐FLAIR fast spin echo, T2* gradient echo echo‐planar, and DWI spin echo echo‐planar).

**Assessment:**

Three radiologists, blinded for clinical information, assessed signs of ischemic lesions (DWI↑, ADC↓, and T2/T2‐FLAIR↑) on EPIMix and routine clinical MRI, with disagreements solved in consensus with a fourth reader to establish the reference standard.

**Statistical tests:**

Diagnostic performance including sensitivity and specificity against the reference standard was evaluated. EPIMix sensitivity was tested for noninferiority compared to the reference standard using Nam's restricted maximum likelihood estimation (RMLE) Score. A *P*‐value < 0.05 was considered statistically significant.

**Results:**

Of 118 patients (mean age 62 ± 16 years, 58% males), 25% (*n* = 30) had MRI signs of acute infarcts. EPIMix was noninferior with 97% (95% CI 83–100) sensitivity for reader 1, 100% (95% CI 88–100) sensitivity for reader 2, and 90% (95% CI 88–98) sensitivity for reader 3 vs. 93% (95% CI 78–99) sensitivity for readers 1 and 2 and 90% (95% CI 74–98) for reader 3 on routine clinical MRI. Specificity was 99% (95% CI 94–100) for reader 1, 100% (95% CI 96–100) for reader 2, and 98% (95% CI 92–100) for reader 3 on EPIMix vs. 100% (95% CI 96–100) for all readers on routine clinical MRI.

**Conclusion:**

EPIMix was noninferior to routine clinical MRI for the diagnosis of acute ischemic stroke.

**Evidence Level:**

2

**Technical Efficacy:**

Stage 2

Diffusion‐weighted imaging (DWI) is the most sensitive MRI sequence for detecting ischemic stroke, with signs of acute brain infarction^1^, ^2^, ^3^, ^4^ appearing only minutes after arterial occlusion.^5^, ^6^ The areas with restricted tissue diffusion demonstrate a high signal on DWI with a corresponding area of low signal on apparent diffusion coefficient (ADC) maps.^7^, ^8^ Subsequently, a high signal on T2 fluid‐attenuated inversion recovery (T2‐FLAIR) images appears in the infarcted area.^9^ Despite MRI having a higher sensitivity in infarction detection,^10^, ^11^, ^12^ computed tomography (CT) remains the primary imaging method for patients with suspected stroke due to its wide availability and fast image acquisition.^13^ Patients with symptoms of acute stroke and initial negative CT and CT angiography (CTA) can then undergo further diagnostic imaging with MRI. In this nonhyperacute stroke work‐up, MRI can confirm a diagnosis of ischemic stroke.^10^

Recently, advances in technology have given rise to several fast MRI protocols, with promising results at imaging times of approximately 5 minutes.^14^, ^15^, ^16^, ^17^ EPIMix is a multicontrast echo‐planar imaging‐based sequence,^18^, ^19^, ^20^, ^21^ which acquires six tissue contrasts (T1‐FLAIR, T2, T2‐FLAIR, T2*, DWI, and ADC) in 78 seconds with only one prescan and without any user interaction or extra time between prescan and scan. EPIMix has shown promise in evaluating patients with suspicion of ischemic infarction in a retrospective observational study at a comprehensive stroke center^21^ but it has not been evaluated in a prospective setting at a primary stroke center. In a regional triaging system, patients with symptoms indicating a high likelihood of large vessel occlusion (LVO) potentially eligible for thrombectomy bypass the primary stroke center,^21^, ^22^ while those with less severe symptoms are directed to a primary stroke center and subject to initial stroke investigation with CT and CTA of the brain. Subsequently, MRI is performed to confirm or exclude a stroke diagnosis in CT‐negative or uncertain cases.

The purpose of this prospective study was to evaluate whether EPIMix was noninferior to routine clinical MRI by evaluating its diagnostic performance for the diagnosis of acute ischemic infarction at a primary stroke center.

## Materials and Methods

### Study Participants and Eligibility

The study was approved by the regional Swedish Ethical Review Authority (approval number/ID 2019‐05741). Written informed consent was obtained from all patients or guardians. In this single‐center prospective noninferiority study, performed at Capio S:t Göran Hospital, a primary stroke center, in Stockholm, Sweden, from February 19 to July 13, 2020 all patients >18 years old with an acute referral to the department of radiology for a brain MR scan due to symptoms of ischemic stroke were invited to participate.

Exclusion criteria were: declined to participate in the study, stroke symptoms lasting >10 days, loss of consciousness, inability to endure the minor time extension required for EPIMix acquisition, radiological inquiries demanding extended MRI work‐up besides stroke, technical issues related to the acquisition of EPIMix or routine clinical MRI, referrals lacking a suspicion of acute ischemia or asking for investigation of area other than the brain.

### Image Acquisition

Scans were performed on a 3‐T clinical MR imaging system (Signa Architect, GE Healthcare, Milwaukee, USA) with a 48‐channel head coil. Routine clinical MRI (T1, T2, T2‐FLAIR, T2*, and DWI) was immediately followed by the EPIMix pulse sequence^18^ acquiring the same contrasts. MRI protocol parameters are listed in Table 1. EPIMix was run with the same 76‐second localizer‐scan as the routine clinical MRI. EPIMix required a single shimming procedure for all six contrasts, while routine clinical MRI required a separate shimming procedure for each of the included sequences. EPIMix reconstruction was automatic with images returned to the MR scanner and picture archiving and communication system after 10 minutes.

**TABLE 1:** MRI Acquisition Parameters

Protocol/Contrast	Scan Time (seconds)	Scan Plane	Matrix Size	Slice t/Gap (mm)	FOV (mm)	Scan Time Without Prescan	Scan Time With Prescan
Localizer	76	3–plane	256 × 128	10/0	300		
EPIMix						1:18	1:37
Echo‐planar T1‐FLAIR	19	Axial	180 × 180	4/0	240		
Echo‐planar T2	12	Axial	180 × 180	4/0	240		
Echo‐planar T2‐FLAIR	12	Axial	180 × 180	4/0	240		
Exco‐planar T2*	6	Axial	180 × 180	4/0	240		
Echo‐planar DWI	23	Axial	180 × 180	4/0	240		
Calibration	6	Axial	180 × 180	4/0	240		
Routine clinical MRI						8:15	9:47
T1 (FSE)	70	Sagittal	260 × 200	4/1	250		
T2 (propeller)	56	Axial	288 × 288	4/0.4	240		
T2‐FLAIR 3D‐fast (turbo) spin echo	218	Sagittal	256 × 256	1.2	256		
T2* GRE EPI	51	Axial	256 × 260	4/0.4	240		
DWI SE EPI	24	Axial	116 × 116	5/0.5	240		

DWI = diffusion‐weighted imaging; FLAIR = fluid‐attenuated inversion recovery; FOV = field of view; FSE = fast spin‐echo; GRE = gradient echo; min = minutes; s = seconds; SE = spin echo; T = Tesla; t = thickness.

### Imaging Assessment

Four readers (R1–R4) performed imaging analysis in PACS, blinded for clinical information including previous radiology work‐up. R1 (S.B.) was a senior radiology resident in‐training with 5 years of radiology experience, R2 (L.L.) a neuroradiologist with 20 years of radiology experience, R3 (F.D.L.) a junior radiology resident in‐training with 2 years of radiology experience and very little experience in clinical brain MRI, and R4 (A.K.) a neuroradiologist with 18 years of radiology experience.

All images were evaluated by R1, R2, and R3 for signs of infarction (DWI hyperintense lesion with a corresponding hypointensity on the ADC map and a hyperintensity on T2/T2‐FLAIR‐weighted images) and classified as acute infarction present or absent. The reference standard for the presence or absence of an acute infarction was defined by a concordant assessment between R1, R2, and R3 on both EPIMix and routine clinical MRI. For cases with discordant infarction assessment between R1, R2 and/or R3, a separate consensus reading with R1, R2, and R4 including side‐by‐side comparisons of EPIMix and routine clinical MRI defined the reference standard. The number and maximum axial diameter of the ischemic lesions as well as their locations were recorded by R1 and R2. Discrepancies in infarction locations between reviewers (R1 and R2) were resolved through consensus. The presence of substantial artifacts was also assessed by R1 and R2. To minimize bias in favor of the new method, EPIMix images were evaluated first with routine clinical MRI assessed after a 13‐week memory‐washout interval. Cases were read in random order.

### Clinical Data

Sociodemographic and medical data of clinical symptoms and cardiovascular risk factors were collected from the information provided in the radiology referral.

### Statistical Analysis

The primary objective of the study was a noninferiority comparison between EPIMix and routine clinical MRI for the diagnosis of acute infarction. Sample size calculation was based on noninferiority for binomial proportions.^23^ The prestudy assumption was that EPIMix would detect at least 95% of the acute ischemic lesions visible on routine clinical MRI set to detect 100% with a noninferiority threshold (i.e. equivalence margin, δ) of 10%. It was estimated that 118 included patients would achieve an 80% power (1‐β) at a significance level of 5% (α) to exclude a difference in favor of routine clinical MRI of more than 10%.^23^ Sensitivity and specificity rates were calculated for R1, R2, and R3 using 2 × 2 contingency tables, and the diagnostic performance of the test method EPIMix and routine clinical MRI against the reference standard was analyzed by receiver operating characteristic (ROC) analysis with the area under the curve (AUC). A pairwise comparison of ROC‐curves for each reader was performed using DeLong's test. Noninferiority testing of EPIMix sensitivity/detection rate compared to the reference standard was performed using the restricted maximum likelihood estimation (RMLE) score of Nam and Blackwelder^24^ at the 5% significance level using a 90% two‐sided confidence interval.

Interobserver agreement was estimated using a linear (kappa) test.^25^ Shapiro–Wilk test was used to test for normal distribution. Results were reported using asymptotic significance 2‐tailed *P* values. Quantitative variables were expressed as means ± SD, or as medians with interquartile range for skewed data. Categorical variables were expressed as counts (percentage).

Statistical analysis was performed using MedCalc for Windows, version 19.6 (MedCalc Software, Ostend, Belgium) and NCSS 2021 Statistical Software (NCSS, LLC. Kaysville, Utah, USA). *P* values < 0.05 were considered statistically significant.

## Results

### Study Population

Out of 147 screened consecutive patients referred for acute MRI, 29 were excluded due to: declining to participate (*n* = 15), symptoms >10 days (*n* = 9), extended radiology work‐up (*n* = 2), no suspicion of acute stroke (*n* = 2), and technical issues (incorrect sagittal imaging plane on EPIMix) (*n* = 1). A total of 118 patients between the age of 19 and 91 (mean 62 ± 16 years, 69 men) were included for further analysis. Patients' characteristics are presented in Table 2. All patients underwent the examination with no adverse events reported as a result of the MRI exams.

**TABLE 2:** Participants' Characteristics

	All Participants (*n* = 118)	Participants With Acute Infarction (*n* = 30)
Age mean (SD) (years)	62 (16)	67 (15)
Sex female, *n* (%)	49 (42)	11 (37)
Cardiovascular risk factors, *n* (%)	64 (54)	18 (60)
Symptoms reported in radiology referral, *n* (%)		
Vertigo/dizziness	48 (41)	9 (30)
Motor dysfunction	33 (28)	15 (50)
Speech difficulties	24 (20)	10 (33)
Sensory dysfunction	24 (20)	5 (17)
Headache	14 (12)	3 (10)
Visual deficits	12 (10)	3 (10)
Diplopia	13 (11)	1 (3)
Confusion/disorientation	9 (8)	2 (7)
Mean delay (±SD), median delay, and range between onset of symptoms and MRI (hours)	74 (±52), 70, 6–240	65 (±43), 48, 12–168
NCCT before MRI	111 (94)	30 (100)

SD = standard deviation;  *n* = number; NCCT = noncontrast computed tomography.

### Primary Outcome

**FIGURE 1:**
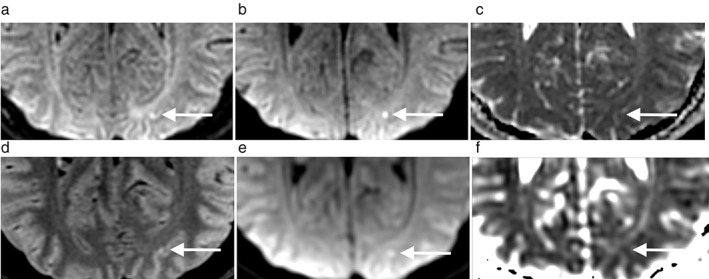
False‐negative finding on routine MRI. A 35‐year‐old man with left vertebral dissection presenting with motor and sensory deficits in left hand and arm. On EPIMix (upper row) DWI (b) shows a 3 mm hyperintense DWI lesion (white arrow) in the left occipital lobe, with a slight hyperintensity on T2‐FLAIR (a) and not visible on the ADC map (c). The suspected lesion is barely visible on reconstructed images of routine clinical MRI T2‐FLAIR (d) and DWI (e) and not visible on ADC (f). The ischemic lesion was detected by both readers on EPIMix but missed on routine MRI.

**FIGURE 2:**
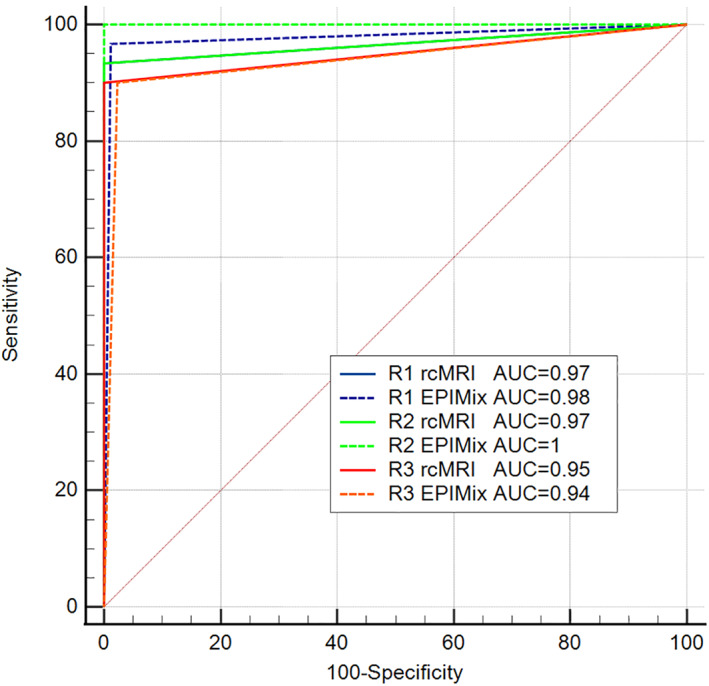
ROC curves. ROC curves comparing EPIMix and routine clinical MR to reference standard for infarction diagnosis. AUC = area under the curve; EPIMix = fast 78‐second multicontrast echo‐planar MR sequence; R1 = reader 1; R2 = reader 2; R3 = reader 3; rcMRI = routine clinical MRI.

Acute infarction was found in 30 of 118 patients (25.4%) according to the reference standard. Assessing EPIMix, R1 and R2 found signs of acute infarction in 30 (25.4%) and on routine clinical MRI in 28 (23.7%) of the patients, R3 on EPIMix in 29 (24.6%) and on routine clinical MRI in 27 (22.9%) of the patients. EPIMix had a total of seven misclassifications (false positive *n* = 1, false negative *n* = 6) and routine clinical MRI had seven (false negative *n* = 7) (Table 3, example in Fig. 1). The sensitivity of EPIMix to detect acute ischemic lesions in comparison to the reference standard was 97% (95% CI 83–100) for R1, 100% (95% CI 88–100) for R2 and 90% (95% CI 88–98) for R3. The specificity of EPIMix was 99% (95% CI 94–100) for R1, 100% (95% CI 96–100) for R2, and 98% (95% CI 92–100) for R3. The sensitivity and specificity for routine clinical MRI were 93% (95% CI 78–99) and 100% (95% CI 96–100) for both R1 and R2. For R3 sensitivity was 90% (95% CI 74–98) and specificity 100% (95% CI 96–100) on routine clinical MRI. Full details on diagnostic performance can be found in Table 4. EPIMix AUC was 0.98 (95% CI 0.93–0.99) for R1, 1.00 (95% CI 0.97–1.00) for R2 and 0.94 (95% CI 0.88–0.98) for R3. Routine clinical MRI AUC was 0.97 (95% CI 0.92–0.99) for both R1 and R2, and 0.95 (95% CI 0.89–0.98) for R3. ROC curves are presented in Fig. 2. Pairwise comparison of ROC‐curves using DeLong's test detected no significant difference between EPIMix and routine clinical MRI (*P* = 0.71, R1, *P* = 0.15, R2, *P* = 0.65, R3). Noninferiority testing (Nam RMLE Score) concluded non‐inferiority for EPIMix sensitivity compared to the reference standard at the 5.0% significance level for both readers (Table 5).

**TABLE 3:** Misclassified Cases

Case	Location	Size (mm)	R1 EPIMix	R2 EPIMix	R3 EPIMix	R1 rcMRI	R2 rcMRI	R3 rcMRI	Consensus Reading and Comment
1	Medulla oblongata right	2	FP	TN	TN	TN	TN	TN	No acute infarction. Artifact on EPIMix classified by R1 as infarction
2	Parietal left	4	FN	TP	FN	TP	TP	TP	Acute infarction. Faint lesions without marked ADC hyposignal classified by R1 as subacute infarcts on EPIMix and missed by R3 on EPIMix
3	Occipital left^a^	2	TP	TP	FN	FN	FN	FN	Acute infarction. Faint lesion with low conspicuity on rcMRI missed by all readers on rcMRI and on EPIMix by R3
4	Frontal left	2	TP	TP	FN	FN	FN	FN	Acute infarction. Faint lesion with low conspicuity on rcMRI missed by all readers on rcMRI and on EPIMix by R3
5	Occipital left	10	TN	TN	FP	TN	TN	TN	No acute infarction. Focal subarachnoid blood in sulci with DWI and T2 FLAIR high signal misclassified by R3 as infarction on EPIMix
6	Mesencephalon left	8	TP	TP	TP	TP	TP	FN	Acute infarction. Infarction with lower conspicuity on rcMRI missed by R3
7	Parietal right	2	TN	TN	FP	TN	TN	TN	No acute infarction. Extraaxial DWI high signal lesion misclassified as infarct by R3 on EPIMix

FN = false negative; FP = false positive; R1 = reader 1; R2 = reader 2; R3 = reader 3; rcMRI = routine clinical MRI; TN = true negative; TP = true positive.

^a^ See Fig. 1.

**TABLE 4:** Sensitivity and Specificity Data

	TP	TN	FP	FN	Sens (95% CI)	Spec (95% CI)	AUC (95% CI)
EPIMix R1	29	87	1	1	96.7% (82.8–99.9%)	98.9% (93.8–100.0%)	0.98 (0.93–0. 99)
EPIMix R2	30	88	0	0	100.0% (88.4–100.0%)	100.0% (95.9–100.0%)	1.00 (0.97–1.00)
EPIMix R3	27	86	2	3	90.0% (88.4–97.9%)	97.7% (92.0–99.7%)	0.94
rcMRI R1	28	88	0	2	93.3% (77.9–99.2%)	100.0% (95.9–100.0%)	0.97 (0.92–0.99)
rcMRI R2	28	88	0	2	93.3% (77.9–99.2%)	100.0% (95.9–100.0%)	0.97 (0.92–0.99)
rcMRI R3	27	88	0	3	90.0% (73.5–97.9%)	100.0% (95.9–100%)	0.95

AUC = area under the curve; CI = confidence interval; FN = false negative; FP = false positive; R1 = reader 1; R2 = reader 2; R3 = reader 3; rcMRI = routine clinical MRI; TN = true negative; TP = true positive.

**TABLE 5:** Results for Sensitivity Noninferiority Testing (Nam RMLE Score) of EPIMix vs. the Reference Standard

	Parameter Tested	PL	Lower 90.0% CL	Upper 90.0% CL	Lower EB	Upper EB	Reject H0 and Conclude Noninferiority at the 5.0% Significance Level
EPIMix R1	Diff (SeEPI − SeRef)	0.0003	−0.03	0.03	−0.1	0.10	Yes
EPIMix R2	Diff (SeEPI − SeRef)	0.0001	−0.02	0.02	−0.1	0.10	Yes
EPIMix R3	Diff (SeEPI − SeRef)	0.0003	−0.03	−0.05	−0.1	0.10	Yes

CI = confidence interval; CL = confidence limit; Diff = difference; EB = equivalency bound; H0 = null hypothesis; PL = probability level (*P* value); R1 = reader 1; R2 = reader 2; R3 = reader 3; SeEPI = sensitivity of EPIMix; SeRef = sensitivity of reference standard.

Lower confidence limit above lower equivalence bound −0.1 indicates noninferior sensitivity of EPIMix to reference standard.

### Secondary Outcomes

**FIGURE 3:**
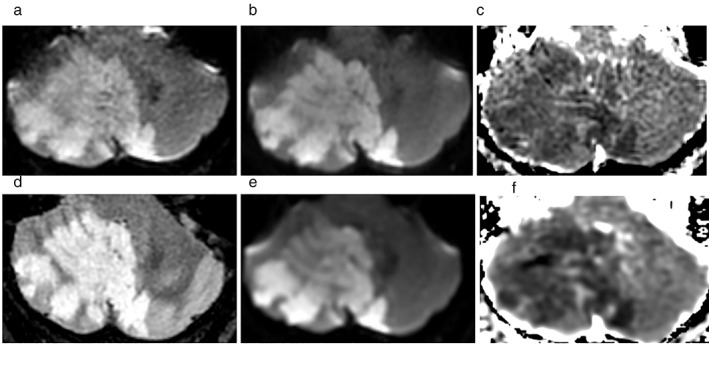
Large ischemic lesion; 49‐year old male with recurrent episodes of vertigo for 4 days and a large acute ischemic lesion in the right cerebellar hemisphere extending into the left hemisphere visible as a hyperintense lesion on EPIMix T2‐FLAIR (a) and EPIMix DWI (b) and as a hypointense lesion on EPIMix ADC (c). The acute infarction is just as visible on the corresponding sequences of routine clinical MRI (d–f).

Interreader agreement for detection or exclusion of acute ischemic lesions was almost perfect for both EPIMix (R1 vs. R2 κ = 0.96, 95% CI 0.89–1.00; R1 vs. R3 κ = 0.89, 95% CI 0.79–0.98; R2 vs. R3 κ = 0.89, 95% CI 0.79–0.98) and routine clinical MRI (R1 vs. R2 κ = 1.00, 95% CI 1.00–1.00; R1 vs. R3 κ = 0.98, 95% CI 0.93–1.00, R2 vs. R3 κ = 0.98, 95% CI 0.93–1.00). The number of acute ischemic lesions per patient ranged from 1 to 15 for EPIMix and 1 to 9 for routine clinical MRI. The median number of acute ischemic lesions in the EPIMix acquisitions was 1 for both readers (interquartile range [IQR] 1–2) and 2 in the routine clinical MRI acquisitions for both readers (IQR 1–2, R1 and 1–3, R2), *P* = 0.40, R1; *P* = 0.76, R2. The maximum diameter of the described lesions ranged from 2 to 66 mm (median 9, IQR 7–14, R1 and 9.5, IQR 6.26–13.75, R2) on EPIMix and from 3 to 57 mm (median 10.5, IQR 7.75–14, R1 and 9, IQR 6.75–13.5, R2) on routine clinical MRI (*P* = 0.19, R1; *P* = 0.08, R2). An example of a patient presenting with a large acute infarct can be seen in Fig. 3. Locations of the acute ischemic lesions (*n* = 30) were supratentorial in 24 (80%), infratentorial in 5 (17%), and both supra‐ and infratentorial in one (3%) patient.

### Artifacts

The presence of substantial artifacts on EPIMIx was described in 7 of the 118 cases (6%). The majority of these (*n* = 5) consisted of susceptibility artifacts, but motion (*n* = 1) and insufficient cerebrospinal fluid saturation (*n* = 1) were also described. For routine clinical MRI, substantial artifacts were described in 12 of the 118 (10%) patients. The majority of these (*n* = 7) consisted of motion artifacts, but ghosting (*n* = 2), wrap (*n* = 1), and susceptibility (*n* = 1) were also reported. No artifact resulted in uninterpretable images.

## Discussion

This prospective study investigating a consecutive patient cohort at a primary stroke center showed noninferiority of a 78‐second multicontrast echo‐planar MRI sequence, EPIMix, compared to routine clinical MRI for the detection of ischemic stroke, with no significant differences in the diagnostic performance between the methods. For noninferiority studies, the goal is to determine whether the new method is noninferior to the one currently in use.^24^, ^26^ Noninferiority threshold for sensitivity defines the range for which the sensitivity of the new method is “close enough” to be considered equivalent and is clinically acceptable. The chosen noninferiority threshold of 10% for EPIMix was based on previously reported sensitivity of 90%–100% of routine clinical MRI for the detection of acute infarcts.^2^, ^4^, ^27^ Interreader agreement was almost perfect despite varying levels of experience between readers. In this study, two of the readers were a radiologist in training and one was a neuroradiologist with 20 years of experience while in a previous report, which showed a similarly high level of agreement,^21^ the readers were neuroradiologists with 15, 15, and 7 years of experience. This study corroborates the findings from a previous retrospective study analyzing a patient cohort in a comprehensive stroke center where the diagnostic performance for acute infarcts was high.^21^

Several other studies have assessed fast brain MRI protocols in emergency settings, with image acquisition times reduced to approximately 5 minutes using techniques such as echo‐planar and parallel imaging, showing good diagnostic performance for the detection of acute infarction.^16^, ^17^ The difference between EPIMix and other MRI protocols is that it requires a single prescan, while other protocols, in general, require a prescan before each sequence and user interaction between sequences with a longer total scan time. Compared to other multicontrast sequences, among these synthetic MRI^28^ and MRI fingerprinting,^29^ the advantage of EPIMix is short acquisition time, simultaneous acquisition of five standard contrast for brain imaging including DWI, and disadvantage lower image resolution.

The routine clinical MRI protocol at our hospital for nonhyperacute work‐up of suspected stroke has an acquisition time of 9.8 minutes. However, reducing acquisition time from 9.8 to 1.6 minutes does not result in a 5‐fold reduction of overall exam time. Beyond scan time, there is the time needed to move the patient on to and away from the table, and localizing procedures that take at least 5–10 minutes depending on the individual patient. Given the reduction in acquisition time and the sequence being robust to motion, EPIMix could be especially useful in the diagnosis of ischemic stroke among patients prone to motion or those who would benefit from a shorter scan time, such as claustrophobic patients, patients suffering from pain or pediatric patients.

Regarding artifacts, there were no uninterpretable studies. However, more susceptibility artifacts were described for EPIMix, because all EPIMix contrasts were echo‐planar imaging based resulting in geometric distortion and signal dropout, especially close to air–tissue or bone–tissue interfaces at the skull base on all contrasts while spin‐echo T1‐weighted, T2‐weighted, and T2‐FLAIR weighted sequences on routine clinical MRI have the advantage of not being affected by susceptibility artifacts. As DWI and T2* images were echo‐planar based with similar artifacts in both methods as previously reported^20^ these artifacts did not cause differences in infarction detection comparing EPIMix and routine clinical MRI. This is in concordance with the previously reported similar diagnostic performance for infarctions in the posterior fossa for EPIMix compared to clinical MRI.^21^ EPIMix had fewer motion artifacts than routine clinical MRI, probably due to the inherent motion‐robustness of the fast single‐shot echo‐planar technique.

### Limitations

Due to the prehospital stroke triaging system^19^, ^22^ described earlier, the main limitation of this study was selection bias toward participants with less severe stroke. The routine clinical MRI protocol is primarily not intended to visualize signs of LVO because CT angiography has already been performed and thus, the routine clinical MRI protocol did not include angiographic sequences. The version of the EPIMix sequence used in this current study did not include angiographic sequences, which is a limitation for evaluation of cases potentially eligible for thrombectomy. However, this is a possible future focus of sequence development.

Further, the lack of quantitative metrics, such as volumes of ischemic lesions, ADC, and contrast‐to‐noise ratios, is a limitation of this study.

Another limitation was the lower resolution of routine DWI compared to EPIMix (matrix size 128 × 128 vs. 180 × 180 and slice thickness 5 vs. 4 mm). Lower lesion conspicuity related to this lower resolution could explain the missed microinfarcts on routine clinical MRI that was detected on EPIMix.

In this nonhyperacute setting, MRI must also rule out differential diagnoses to acute ischemic stroke such as brain hemorrhage, status epilepticus, encephalitis and brain tumors, which motivates the inclusion of all contrasts in EPIMix. This has been the focus of a previous study evaluating EPIMix^19^ but was not the focus of the current study.

Further, this was a single center study conducted on one MR scanner from one manufacturer with one field strength (3 T). Despite using a different scanner, hospital and patient setting compared to a previous report,^21^ EPIMix results were repeatable and thus support generalizability.

## Conclusion

In conclusion, this prospective study showed that a 78 seconds EPIMix acquisition was noninferior to routine clinical MRI for the diagnosis of acute ischemic stroke with high diagnostic performance and high agreement between readers.
